# Effect of Large-Scale Paddy Rice Drying Process Using Hot Air Combined with Radio Frequency Heating on Milling and Cooking Qualities of Milled Rice

**DOI:** 10.3390/foods11040519

**Published:** 2022-02-11

**Authors:** Karn Chitsuthipakorn, Sa-nguansak Thanapornpoonpong

**Affiliations:** 1 Postharvest Technology Research Center, Faculty of Agriculture, Chiang Mai University, Chiang Mai 50200, Thailand; karn_c@cmu.ac.th; 2 Department of Plant and Soil Sciences, Faculty of Agriculture, Chiang Mai University, Chiang Mai 50200, Thailand

**Keywords:** paddy, rice, hot air drying, radio frequency heating, milling quality, cooking quality

## Abstract

The objectives of the study on a continuous flow hot air dryer combined with radio frequency heating at different temperatures (HA/RF) (38 °C, 42 °C, 46 °C, and 50 °C) in a large-scale process compared with conventional continuous flow hot air drying (HA) were (1) to investigate the drying characteristics, drying kinetics, and milling quality of the process and (2) to observe the cooking quality and compare the sensory differences of the cooked rice after treatment. The drying characteristics and moisture diffusivity showed that the higher the radio frequency (RF) heating temperature, the shorter the drying time. The specific energy consumption and energy cost decreased when the RF heating temperature increased. The optimal condition in terms of fissure percentage was HA/RF42. In addition, there were no significant differences in head rice yield and white rice color determination, amylose content, texture profiles, and pasting properties in all HA/RF treatments. In the triangle test, it was found that at least 6% of the population could perceive a difference between HA and HA/RF50. In conclusion, this study proposes the further development of the HA/RF drying process at low-temperature profiles and shows the great potential of RF technology for commercial drying in rice industry.

## 1. Introduction

Paddy rice cultivation plays an important role in the Thai economy. About 30 million tons of paddy rice are harvested annually. It is usually harvested with a combine harvester at a moisture content of 18–28% (w.b.) and immediately dried to below 14% for safe storage. The most common large-scale mechanical drying systems used in Thailand are continuous flow dryers and recirculating batch dryers, as they offer advantages in terms of cost and drying speed. Paddy is dried with hot air (HA) at a temperature of (50 °C–80 °C) to achieve the shortest drying time because, the shorter the drying time, the more the business turnover can be raised.

Radio frequency (RF) heating is a new type of processing technology that uses electromagnetic waves in the frequency range of 1–300 MHz [1]. It has recently been used for paddy drying [2], accelerated aging [3,4,5], paddy seed treatment [2], Aspergillus flavus control [6], and insect control in milled rice [7,8,9,10]. RF heating, as a type of dielectric heating method, involves the generation of heat as a result of the interaction between an electromagnetic field and polarized molecules in the crops, which are either bipolar or ionic [11]. In hot air drying combined with the RF heating application, the paddy is placed between two electrodes, causing the polar molecules in the paddy to rotate and generate heat energy throughout the paddy volume; it also penetrates the products and generates heat uniformly and instantaneously in the paddy kernel without regard to convective or conductive media. The heat generated promotes evaporation of moisture from the paddy surface and stimulates moisture migration from the inside to the outside. The most successful past applications have often combined two or more technologies (RF–HA, etc.). Most notably, the use of hot air (HA) in combination with RF heating to dry paddy has been investigated to develop a commercial dryer for the rice processing industry with a lower carbon footprint in the future.

Based on the vertical operating prototype that supported an RF heating system by Vearasilp et al. [10] and the earlier study by Chitsuthipakorn and Thanapornpoonpong [12], the electrical voltage control of the RF heating machine was set manually, which proved to be a disadvantage for the authors. There were two different ways to solve this problem: (1) installing an automatic system with a vibration feeder to control the paddy release rate from the RF heating machine to control the paddy temperature in the RF heating chamber, and (2) installing an automatic system to control the electrical voltage to control the RF heating energy for the paddy. After many experiments, it was found that installing an automatic system to control the electrical voltage resulted in a constant paddy temperature in the RF heating chamber. Therefore, the authors decided to conduct the study with a lower temperature in a narrower range. In addition, the lowest temperature of the study was 38 °C because it was close to the ambient temperature, which was about 30–35 °C. Likewise, the optimal condition in the previous study was at the lowest RF heating temperature (HA/RF45) in the experimental design, and it may be too early to conclude that HA/RF45 is the appropriate condition for this improved system. The prototype machine in this study was upgraded with respect to the RF heating chamber by replacing a Polypropylene (PP) chamber with a Polytetrafluoroethylene (PTFE) chamber because it is nonstick, nonwetting, the United States Food and Drug Administration (FDA) compliant, and resistant to high temperatures, and by using an automatic RF heating temperature control system. The specific objectives of the study of the hot air dryer combined with RF heating in a large-scale process compared to conventional hot air drying were (1) to investigate the drying characteristics, drying kinetics, and milling quality of the process and (2) to observe the cooking quality and compare the sensory differences of the cooked rice after treatment.

## 2. Materials and Methods

### 2.1. Materials

The study used a randomized complete block design (RCBD) with three replicates. A continuous flow hot air dryer was used in combination with different radio frequency (RF) heating temperatures measured at paddy in the RF heating chamber (38 °C, 42 °C, 46 °C, and 50 °C). A total of 9000 kg of freshly harvested paddy rice (variety RD 41) was purchased from local farmers in Nakhon Sawan province, Thailand, in November 2021. Initial moisture content averaged 25–26% (w.b.). It was stored in PP-woven bags separated for each replicate (450 kg paddy/bag). To avoid quality deterioration, each replicate was treated within 4 days after harvest. After treatment, 25 kg paddy samples from each replicate were stored for quality determination.

### 2.2. Hot Air Dryer and RF Heating Systems

**Figure 1:**
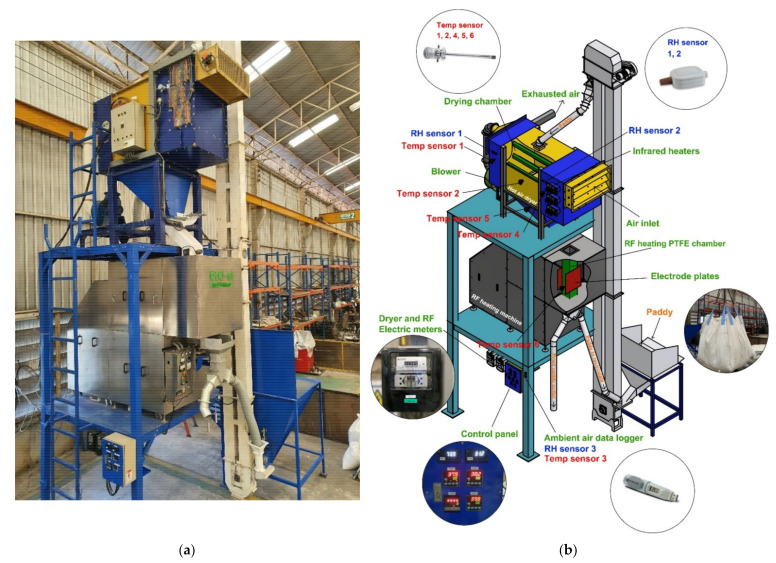
The industrial-scale continuous-flow hot-air dryer with RF heating machine: (**a**) prototype machine (**b**) schematic diagram.

Figure 1a shows the industrial-scale continuous flow hot air dryer with an RF heating machine that was used in this study. It mainly consists of a recirculating hot air dryer (QS -500, Quaser Engineering, Pathum Thani, Thailand) capable of drying up to 450 kg of paddy at a time, a 15 kW, 27.12 MHz RF heating machine (BiO-Q model S-1, Yont Phol Dee, Nakhon Sawan, Thailand), and a bucket elevator. The electrode gap of RF heating was 170 mm, with the length and width of the electrode plate to be 450 mm and 300 mm. The RF energy was regulated to produce the desired paddy temperatures by automatically increasing or decreasing the electrical voltage on the control panel of the machine. The voltage supplied to the RF heating machine was adjusted by a variable autotransformer (Variac) to stabilize the RF heating temperature for paddy. The maximum electrical input voltage was 380 V, and the voltage used in HA/RF38, HA/RF42, HA/RF46, and HA/RF50 was 90–100 V, 100–140 V, 120–180 V, and 150–220 V, respectively. Air is circulated from a blower (2 HP/2850 rpm/28 CMM/80 mmAq) at the rear of the dryer through the heating chamber at the front, which contains three rows of 1000-watt infrared heaters. The hot air temperature was regulated at the beginning and automatically controlled by a sensor and control device during drying. The hot air from the drying section circulated through the paddy, with evaporated water being extracted by a blower and released to the environment. As shown in Figure 1b, the dryer is equipped with five temperature sensors (PT100, Primus Thai, Bangkok, Thailand), two relative humidity sensors (RHM, Primus Thai, Bangkok, Thailand), and one humidity and temperature data logger (EL-USB-2-LCD, Lascar Electronics Inc., Erie, PA, USA). Fresh paddy was dumped into the intake pit and transported to a hot air dryer by a bucket elevator. To maintain the quality of the milled rice, the hot air temperature was manually adjusted with a temperature controller and divided into four different levels: 80 °C at a moisture content of 25–26%, 70 °C at a moisture content of 20–25%, 60 °C at a moisture content of 17–20%, and 50 °C at a moisture content of 13–17%. Hot air was blown through the paddy in the drying chamber while the paddy was continuously conveyed from the hopper into the RF heating machine. As the paddy moved through the RF heating chamber, it was heated to the desired temperature (38 °C, 42 °C, 46 °C, and 50 °C) using the required RF energy. A temperature sensor 6 (PT100, Primus Thai, Bangkok, Thailand) was placed in the RF heating chamber to monitor the temperature of the paddy after heating. After the paddy was discharged from the RF heating machine into the bucket elevator, it was conveyed through a vibratory feeder controlled by a fixed input voltage electrical control. The average flow rate of the recirculation was 494 kg of paddy/hour. This process was repeated continuously and not in batches until the moisture content of the paddy fell below 14% (w.b.).

### 2.3. Moisture Content Determination

Moisture content was measured in triplicate every 30 min throughout the drying process using a rapid capacitance-type moisture meter (Granomat, Pfeuffer GmbH, Kitzingen, Germany) to observe the value of moisture content during the drying process, which was reported as mean and standard deviation.

### 2.4. Drying Efficiency

#### 2.4.1. Drying Kinetics

Because the initial moisture content of paddy was not identical for all treatments, the moisture content data were converted using Equation (1) to obtain the dimensionless moisture ratio. Over time, the equilibrium moisture content (M_e_) becomes insignificant and relatively small compared with the moisture content of paddy at each time point (M_t_) and the initial moisture content of paddy (M_0_) [13,14], as prolonged exposure of the grain to infrared radiation eventually causes the material to burn [15]. The moisture ratio (MR) can be simply expressed as follows:

(1) $$\mathrm{MR}=\frac{M_{t}-M_{e}}{M_{0}-M_{e}}$$

The drying kinetics of this study were described using the Lewis model and the Henderson–Pabis model, as shown in Equations (2) and (3). These models are based on Newton’s law of cooling and Fick’s second law of diffusion, respectively.

(2) $${\mathrm{MR}}_{\mathrm{LW}}=e^{-\mathrm{kt}}$$

(3) $${\mathrm{MR}}_{\mathrm{HP}}={a\;e}^{-\mathrm{kt}}$$

where MR_LW_ denotes moisture ratio using the Lewis model, MR_HP_ denotes moisture ratio using the Henderson–Pabis model, a denotes model constant, k denotes rate constant, and t is drying time in minutes. The rate constant (k) represents drying performance, with a high value representing a fast-drying rate [16].

#### 2.4.2. Effective Moisture Diffusivity

Using Equations (4)–(6), the effective moisture diffusivity was derived to characterize moisture movement in paddy during the drying process [17].

(4) $$\mathrm{MR}=\frac{6}{\pi^{2}}\exp\left(-\frac{\pi^{2}D_{\mathrm{eff}}t}{R_{s}^{2}}\right)$$

where D_eff_ is the effective moisture diffusivity (m^2^/s), t is the drying time in minutes, and $R_{s}^{2}$ is the equivalent radius of the sphere (m). Equation (4) evaluated numerically for Fourier number (F_o_) given by ($D_{\mathrm{eff}}t/R_{s}^{2})$ for diffusion and can be rewritten as:

(5) $$\mathrm{MR}=\frac{6}{\pi^{2}}\exp\left(-\pi^{2}\mathrm{Fo}\right)$$

(6) $$\mathrm{Fo}\;=-0.1013\ln\left(\mathrm{MR}\right)-0.0504$$

(7) $$D_{\mathrm{eff}}=\left(\frac{\mathrm{Fo}}{t/R_{s}^{2}}\right)$$

where D_eff_ was estimated by substituting the positive values of F_o_ and the drying time (t), along with the equivalent radius of paddy grain in Equation (7). The paddy grain is approximated as isotropic spheres of 3 mm diameter [17].

#### 2.4.3. Energy Consumption

A specific energy consumption (SEC) of the drying was determined using Equation (8) [18]:

(8) $$\mathrm{Specific}\;\mathrm{energy}\;\mathrm{consumption}\;\left(\mathrm{SEC}\right)=\frac{E_{\mathrm{total}}}{m_{\mathrm{eva}}}$$

where SEC is the specific energy consumption (MJ/kg-H_2_O), E_total_ is the total electrical energy supplied to the dryer and RF heating systems during the drying process (kWh), and m_eva_ is the amount of evaporated water resulting from the difference in weight of the paddy before and after drying (kg-H_2_O). Considering that the cost of electricity in Thailand was THB 3.2484 per kWh and the exchange rate THB/USD was 33.15:1 on 3 January 2022, the energy cost per kg of evaporated water (USD/kg-H_2_O) was estimated using Equation (9):

(9) $$\mathrm{Energy}\;\cos t=\frac{\left(P_{\mathrm{dryer}}+P_{\mathrm{rf}}\right)}{m_{\mathrm{eva}}}\times \frac{3.2484}{33.15}$$

where P_dryer_ is the power supplied to the dryer system, and P_rf_ is the power supplied to the RF heating system.

### 2.5. Milling Quality Evaluation

#### 2.5.1. Fissure Percentage

The dried paddy samples were cleaned and dehusked to obtain brown rice. One hundred kernels (about 2 g) were taken at random for each measurement. The measurement was performed three times for each replicate, and the mean and standard deviation were calculated. The brown rice samples were placed on a transparent plate over a fluorescent tube and a magnifying glass to visually count the fissured kernels [19]. The fissures in the kernels of brown rice were examined with the naked eye and recorded with a fissure degree of 0, 1, 2, 3, 4, and 5 or more fissures [20]. According to Shen et al. [20], the fissure percentage was introduced to quantitatively calculate the proportion of brown rice kernels with different fissure degrees. It was defined as the ratio of brown rice kernels with different fissure degrees to the total amount of selected kernels. Equation (10) illustrates the procedure for calculating the fissure percentage.

(10) $$\mathrm{Fissure}\;\mathrm{percentage}\;\left(\%\right)=\frac{N_{f}}{N_{t}}\times 100$$

where N_f_ is the total amount of brown rice kernels at a certain fissure degree (0, 1, 2, 3, 4, ≥5), and N_t_ is the total amount of the brown rice kernels.

#### 2.5.2. Milling Yield

The milling yield was determined according to the method described in the Thai Standards for Rice [21]. A precleaning machine was used to clean the dried paddy (TPC-05, Yont Phol Dee Co., Ltd., Nakhon Sawan, Thailand). A total of 375 g of clean paddy from each replicate was collected, divided into 125 g portions, and stored in a zippered plastic bag. The measurement was repeated three times, and the mean and standard deviation were calculated. The weight of brown rice was determined after it was dehusked twice using a rubber roller husking machine (THY-05, Yont Phol Dee Co., Ltd., Nakhon Sawan, Thailand). The brown rice was then milled for 20 s in a horizontal friction-type milling machine (TFW-05, Yont Phol Dee Co., Ltd., Nakhon Sawan, Thailand). The white rice was collected, and its weight was determined. Finally, the broken white rice was sorted and separated from the head rice using a circular perforated sieve (diameter 2.4 mm) and manual picking to ensure that the broken rice was completely separated from the head rice. The head and broken rice were weighed. After the weight data were recorded, the yields of husk, bran, and head rice were calculated using Equations (11)–(13).

(11) $$\mathrm{Husk}\;\mathrm{weight}\;\mathrm{percentage}\;\left(\%\right)=\frac{W_{\mathrm{pd}}-W_{\mathrm{br}}}{W_{\mathrm{pd}}}\times 100$$

(12) $$\mathrm{Bran}\;\mathrm{weight}\;\mathrm{percentage}\;\left(\%\right)=\frac{W_{\mathrm{br}}-W_{\mathrm{wr}}}{W_{\mathrm{pd}}}\times 100$$

(13) $$\mathrm{Head}\;\mathrm{rice}\;\mathrm{weight}\;\mathrm{percentage}\;\left(\%\right)=\frac{W_{\mathrm{hr}}}{W_{\mathrm{pd}}}\times 100$$

where W_pd_ is the weight of rice from each measurement, W_br_ is the weight of brown rice, W_wr_ is the weight of white rice, and W_hr_ is the weight of the head rice yield.

#### 2.5.3. White Rice Color Determination

The color value of white rice was determined with the CIE (L*, a*, b*) color scale using a chromameter (CR-400, Konica Minolta Sensing, Osaka, Japan). The whiteness index (WI) was used to calculate and compare the whiteness level of rice, as shown in Equation (14).

(14) $$\mathrm{Whiteness}\;\mathrm{index}\;\left(\mathrm{WI}\right)=100-\sqrt{\left[{\left(100-L*\right)}^{2}+{a*}^{2}+{b*}^{2}\right]}$$

where L* is lightness–darkness (0 ≤ L ≤ 100), a* is redness in a positive value, a* is greenness in a negative value, b* is yellowness in a positive value, and b* is blueness in a negative value. The total color difference (ΔE*) used in repeatability testing to serve as a single number for pass/fail decision was defined in Equation (15).

(15) $$\mathrm{Total}\;\mathrm{color}\;\mathrm{difference}\;\left({\Delta E}^{*}\right)=\sqrt{{\left(\Delta L*\right)}^{2}+{(\Delta a*)}^{2}+{(\Delta b*)}^{2}}$$

where ΔL* is the L* sample minus L* reference, Δa* is the a* sample minus a* reference, and Δb* is the b* specimen minus b* reference. For each replicate, the measurement was repeated three times and reported as the mean and standard deviation.

### 2.6. Cooking Quality Evaluation

#### 2.6.1. Elongation Ratio

Each treatment was repeated three times, and the mean and standard deviation were calculated. In each replicate, 50 white rice kernels were randomly drawn from the samples. Rice was soaked in water for 30 min before being placed on a strainer to boil. After 10 min of boiling, rice was spread on a plate, and the 30 kernels of rice grains that were elongated straightly were measured. The elongation ratio was calculated by dividing the average length of the cooked rice by the average length of the uncooked rice. The elongation ratio was calculated as follows [22]:

(16) $$\mathrm{Elongation}\;\mathrm{ratio}=\frac{\mathrm{Average}\;\mathrm{length}\;\mathrm{of}\;\mathrm{cooked}\;\mathrm{rice}\;\left(\mathrm{mm}\right)}{\mathrm{Average}\;\mathrm{length}\;\mathrm{of}\;\mathrm{uncooked}\;\mathrm{rice}\;\left(\mathrm{mm}\right)}$$

#### 2.6.2. Apparent Amylose Content

The method specified in the Thai Standards for Rice [21] was used to determine the apparent amylose content of rice samples using a spectrophotometer (SPECORD 40, Analytik Jena, Jena, Germany) at a wavelength of 620 nm. Each treatment was repeated three times, and the results were expressed as mean and standard deviation.

#### 2.6.3. Texture Profiles Analysis

The milled rice was washed and cooked with a rice-to-water weight ratio of 1:1.7. It was cooked to completion in 1 L household rice cookers, followed by a 10 min warming period. The cooked rice was taken directly from the center of each pot for testing. A texture analyzer (TA.XT plus, Stable Micro Systems, Surrey, UK) was used to analyze texture profiles according to the method of Champagne et al. [23]. On a glass plate, 10 cooked rice kernels were arranged in a single grain layer. A compression plate was set at the height of 5 mm above the base. To allow the plate to travel 4.9 mm, return, and repeat the test, a two-cycle program for compression, force, and distance was used. The test speed was 1 mm per second. A cylinder plunger with a diameter of 50 mm was used. Each treatment was repeated three times, and results were reported as mean and standard deviation.

#### 2.6.4. Pasting Properties

Rice viscosity (RVA profiles) was determined using a rapid viscosity analyzer (RVA-4, Newport Scientific, New South Wales, Australia) according to AACC Method 61-02, Determination of the Pasting Properties of Rice with the Rapid Visco Analyzer, and analyzed using Thermal Cycle (TCW 2.5) software for Windows [24]. Three grams of flour from each sample was weighed into an aluminum canister at a moisture content of 14%, to which 25 mL of distilled water was added. A paddle was placed in the canister, and its blade was vigorously jogged up and down through the sample ten times. The RVA dispersed the samples by rotating the paddle at 960 rpm for the first 10 s of the test. Viscosity was then measured at a constant paddle speed of 160 rpm. The idle temperature was set at 50 °C, and the following 12.5 min test profiles were performed: (1) 50 °C was held for 1.0 min, (2) the temperature was linearly raised to 95 °C until 4.8 min, (3) the temperature was held at 95 °C until 7.5 min, (4) the temperature was linearly reduced to 50 °C until 11 min, and (5) held at 50℃ until 12.5 min. Heating and cooling were performed linearly between the profile set points. Each treatment was repeated three times, and the results were expressed as mean and standard deviation.

### 2.7. Sensory Analysis

The triangle test was performed in accordance with the requirements of ISO 4120:2004. It was conducted to determine whether panelists, who were 24 untrained panelists who normally consumed rice on a regular basis, could distinguish the difference between cooked rice treated with RF combined hot air drying and cooked rice treated with hot air drying. Rice was washed and boiled at a rice-to-water weight ratio of 1:1.7. Rice was cooked to completion in 1 L household rice cookers, followed by a 10 min warming period. The cooked rice (1.5 g) was then served at 60 °C in a white plastic bowl with a 3-digit coding. An equal number of the six possible sequences of products A and B: ABB, AAB, ABA, BAA, and BAB were randomly distributed to panelists in groups of 6 until 24 panelists had completed the evaluation. The perceptible difference was determined using the standardized tables from ISO 4120:2004 [25].

### 2.8. Statistical Analysis

All experimental data were presented in terms of mean ± standard deviation (SD). Analysis was performed by analysis of variance (ANOVA) and Duncan’s new multiple range test (DMRT) for comparison of means at the 5% probability level (*p* < 0.05) using SPSS software version 25 (IBM, New York, NY, USA).

## 3. Results and Discussion

### 3.1. Drying Characteristics

#### 3.1.1. Drying Model

**Figure 2:**
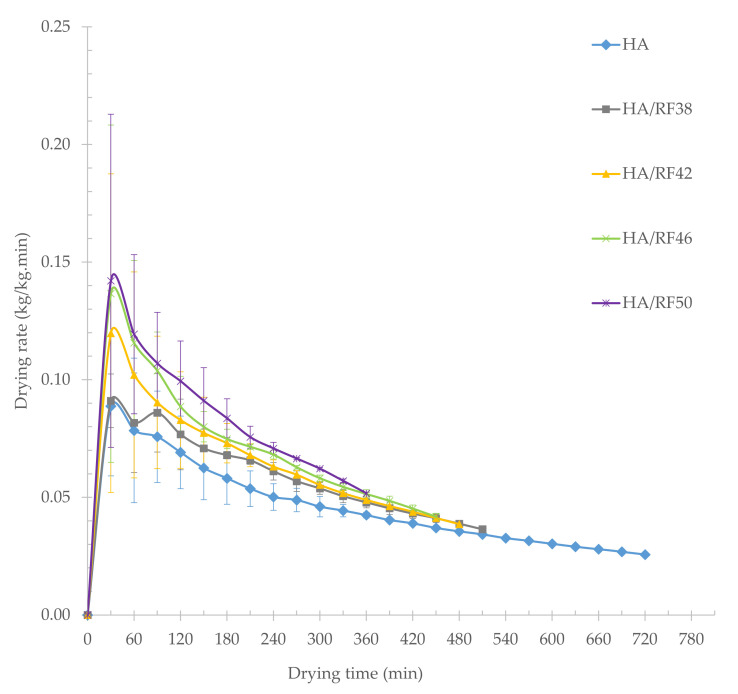
Drying rate versus drying time for paddy dried with hot air and hot air combined with various RF heating temperatures.

**Figure 3:**
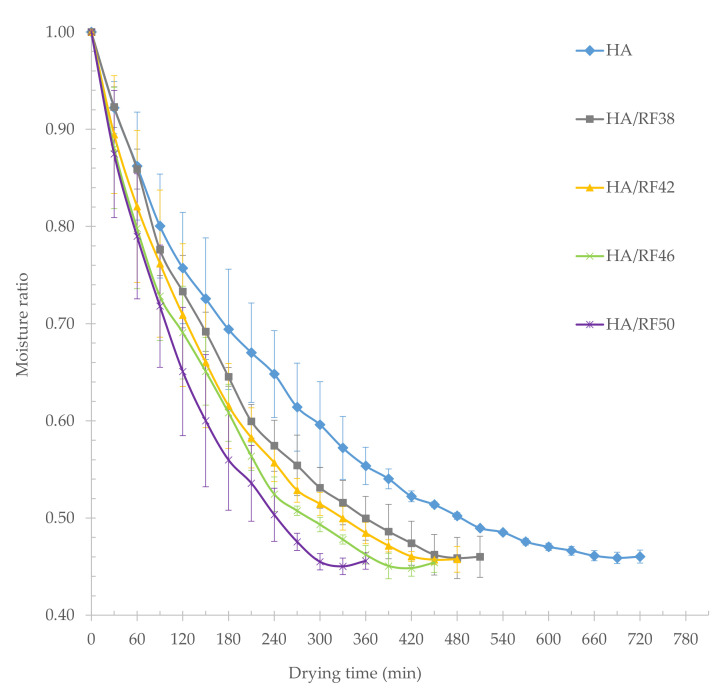
Drying kinetics for paddy dried with hot air and hot air combined with various RF heating temperatures.

Figure 2 illustrates the drying rate versus the drying time for paddy dried with hot air and hot air combined with various RF heating temperatures. During the first 30 min of drying, the drying rate by hot air (HA), hot air with RF heating temperature of 38 °C (HA/RF38), hot air with RF heating temperature of 42 °C (HA/RF42), hot air with RF heating temperature of 46 °C (HA/RF46), and hot air with RF heating temperature of 50 °C (HA/RF50) were 0.0887, 0.0910, 0.1197, 0.1366, and 0.1421 kg water/kg dry matter per minute, respectively. The moisture ratio versus time for paddy dried with hot air and hot air combined with various RF heating temperatures is shown in Figure 3. After the first 30 min of drying, the moisture ratio of paddy dried by HA, HA/RF38, HA/RF42, HA/RF46, and HA/RF50 were 0.9221, 0.9228, 0.8945, 0.8807, and 0.8745, respectively. Following that, the curves demonstrated exponential decay.

At a final moisture content of 14%, drying paddy at HA, HA/RF38, HA/RF42, HA/RF46, and HA/RF50 took about 720, 510, 480, 450, and 360 min, respectively. Compared with HA drying time as the baseline value, the savings of drying time were 29.17%, 33.33%, 37.50%, and 50.00%, respectively. As a result, the drying time required for combined drying with HA/RF was advantageously shorter than that required for drying with HA. In all treatments, it was found that drying time was shortened when RF heating temperature increased because, in the early drying stage, the moisture content of fresh paddy was 25–26% (w.b.). The high water content in the paddy absorbed more energy at different moisture contents due to the dielectric properties of paddy seed in different moisture contents [2], and free water with high water activity accounted for most of the water content in the paddy at this early stage. The transfer and accumulation of heat accelerated the evaporation of free water in the paddy. This may indicate that the drying rate depends on the radiation intensity, which was exerted by the RF heating temperature in this study, and that the drying time was shortened with increasing radiation intensity [26,27].

The estimated values of the coefficients for selected thin-layer drying models of combined RF heating with hot air drying are shown in Table 1. In the Lewis and Henderson–Pabis models, the values of drying rate constant (k) as a function of RF heating temperature ranged between 1.3405 × 10^−3^ to 2.6657 × 10^−3^ and 1.0373 × 10^−3^ to 2.2164 × 10^−3^, respectively. The coefficients of determination (R^2^) of the Lewis model fit the curves better than the R^2^ of Henderson–Pabis model, which were 0.9702–0.9776 and 0.9286–0.9420, respectively. Thus, the Lewis model was suitable for predicting the drying characteristics observed in the study. Moreover, the values of the drying rate constant (k) were found to increase with increasing RF heating temperature.

#### 3.1.2. Effective Diffusivity

The average effective moisture diffusivity (D_eff_) of the combined RF heating with hot air drying is shown in Table 1. The study found that the D_eff_ varied between 4.8156 × 10^−3^ to 9.4426 × 10^−3^ and raised with increasing RF heating temperature. This could be explained by the fact that the energy of RF affected the rapid temperature rise of the paddy kernels from the inside to the outside, which increased the vapor pressure and consequently accelerated the diffusion of moisture to the surface [28].

#### 3.1.3. Energy Consumption

Table 2 shows the electricity consumption of the drying system, including the hot air dryer and the RF heating machine, the specific energy consumption (SEC), which indicates the amount of energy consumed (MJ) to evaporate the water in the paddy (kg-H_2_O), and the specific energy cost, which indicates the electricity cost (USD) to evaporate the water per kilogram (kg-H_2_O). The amount of SEC and the specific energy cost decreased at all RF heating temperatures, with HA/RF50 having the lowest values for water evaporation of 3.8600 ± 0.2261 MJ/kg-H_2_O and 0.1009 ± 0.0121 USD/kg-H_2_O, respectively. Olatunde et al. [29] reported that the specific energy consumption of 4.574–4.905 MJ/kg-H_2_O was required for drying paddy rice in one pass from an initial moisture content of 24% to 11–13% using the MW dryer, and [30] also reported that 4.04 kWh/kg-H_2_O (14.54 MJ/kg-H_2_O) was required for drying parboiled paddy rice from an initial moisture content of 55.96% to 15.58% using the MW dryer. In this study, the SEC and specific energy costs showed no significant difference, while the electricity consumption of the hot air dryer decreased significantly but that of the RF heating machine increased significantly when the RF heating temperature increased. This might indicate that the power of RF, together with the power of the hot air dryer, helps to evaporate a certain amount of water from the paddy with a certain SEC. Therefore, this could be a useful observation that drying paddy with the method presented in this study requires less energy than drying with the MW dryer, as mentioned above.

### 3.2. Effects on Milling Qualities

#### 3.2.1. Fissure Percentage

**Figure 4:**
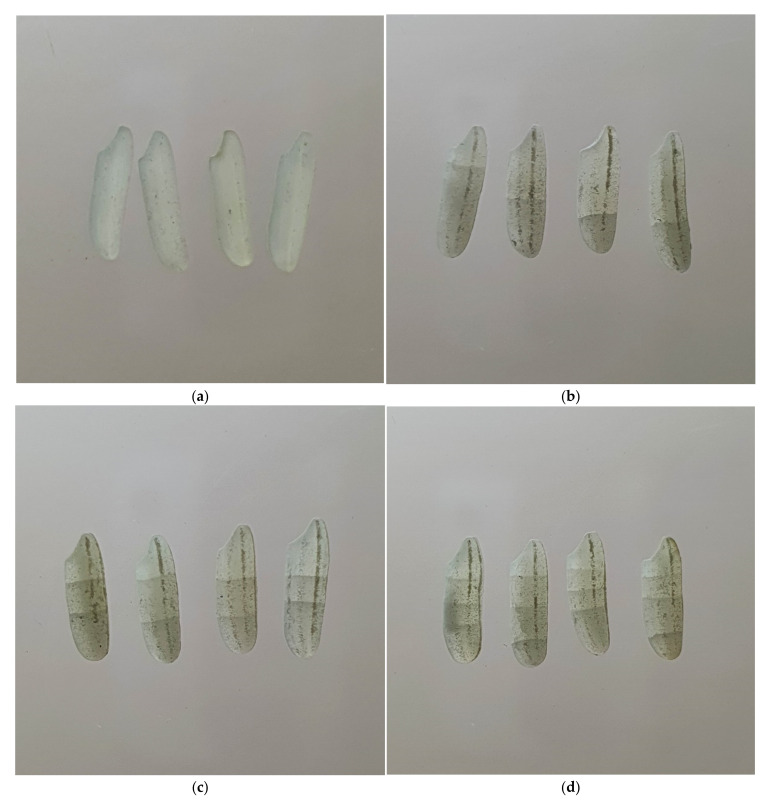

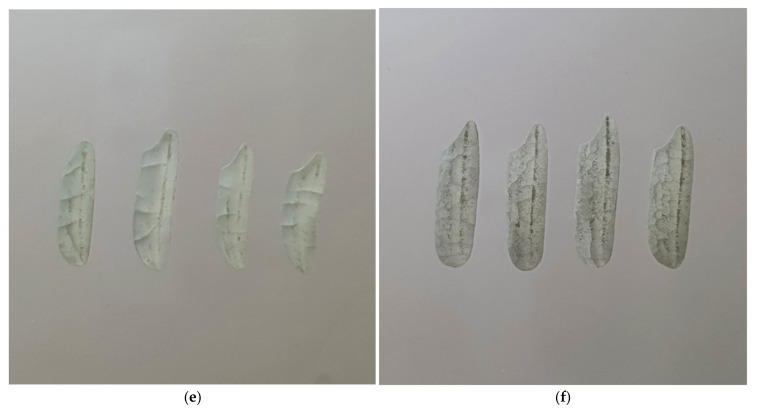
Morphology of different fissure degrees in white rice: (**a**) white rice with 0 fissure; (**b**) white rice with 1 fissure; (**c**) white rice with 2 fissures; (**d**) white rice with 3 fissures; (**e**) white rice with 4 fissures; (**f**) white rice with ≥5 fissures.

The effects of different RF heating temperatures on the fissure percentage of brown rice with different fissure degrees are shown in Table 3. The fissure percentage was classified into four groups, namely None (0 fissure), Few (1–2 fissures), Moderate (3–4 fissures), and Severe (≥5 fissures), as reported by [31]. The morphology of fissures in white rice with different fissure degrees is shown in Figure 4. The fissure percentage of brown rice kernels with “None” fissure degree was significantly decreased from 99.78% ± 0.44% to 85.33% ± 1.87% from HA to HA/RF50. In contrast, the fissure percentages of brown rice kernels with “Few”, “Moderate”, and “Severe” fissure degrees were significantly increased from 0.33% ± 0.50% to 12.44% ± 1.81%, 0.00% to 0.56% ± 0.73%, and 0.00% to 1.78% ± 0.83% from HA to HA/RF50, respectively. The “None” fissure degree considered the most important characteristic, while the “Severe”, “Moderate”, and “Few” fissure degrees considered the most undesirable characteristic, respectively, because fewer fissure kernels would result in a high head rice yield of milled rice. This result showed that RF heating significantly affected the fissure percentage due to the high absorption of RF energy inside the rice kernels [31] and that the temperature of the rice grain plays an important role in the development of a heat and moisture gradient inside the grain, resulting in surface hardening [32] and differential stress [33], respectively. The optimum condition for RF heating temperature was HA/RF42, as no “Moderate” and “Severe” fissure degrees were observed. Referring to the earlier study by Chitsuthipakorn and Thanapornpoonpong [12], drying paddy with hot air combined with RF heating temperatures of 45 °C, 50 °C, 55 °C, and 60 °C showed that the critical limit of RF heating temperature was HA/RF45 because the least “Moderate” and “Severe” fissure degrees were observed. Moreover, the fissure percentage result was relatively lower in this study because of the modification of the prototype machine, as mentioned in the Introduction.

#### 3.2.2. Milling Yield

The milling yield percentage determination after drying at different RF heating temperatures is shown in Table 4. It was found that the husk, brown rice, and broken rice yields significantly increased after treatment and ranged from 24.62 ± 0.78 to 25.78 ± 0.67, 74.22 ± 0.67 to 75.38 ± 0.78, and 26.22 ± 1.37 to 28.00 ± 1.79, respectively. However, head rice yield (HRY) is the most important characteristic among industrial rice millers because it has a proportional effect on the income of the enterprise, while husk and broken rice are less important because of their lower price per kg, which is about 50% of the head rice price. Thus, the higher the head rice yield, the better the drying conditions for rice millers. In this study, there was no significant difference in HRY, which could mean that all RF heating temperatures were suitable for large-scale paddy drying. With reference to the previous study by Chitsuthipakorn and Thanapornpoonpong [12], drying paddy with hot air combined with RF heating temperatures of 45 °C, 50 °C, 55 °C, and 60 °C showed that the critical limit of RF heating temperature was HA/RF50 in terms of HRY and the optimum condition was HA/RF45 in terms of HRY, color, and fissure percentage. Therefore, the aim of this study was to achieve the most suitable drying conditions by combining hot air and RF heating through a lower RF heating temperature and a narrower temperature level. Similarly, the development of a one-pass microwave heating for paddy drying was recently investigated and found that the application of microwave energy of up to 600 kJ/kg-grain to medium-grain paddy, with an initial moisture content of 23% to 24% and an additional tempering step at 60 °C for 4 h, dried the paddy to a final moisture content of 14% to 16%, depending on the rate of energy application, with HRY not significantly different from natural air at 25 °C and RH of 65% [27].

#### 3.2.3. White Rice Color Determination

Table 5 shows the evaluation of the color of white rice after drying at different RF heating temperatures. It was found that the white rice had a slightly greenish-yellowish color after treatment. There was only a significant difference in the yellow-blue value (b*), while the lightness value (L*) and the red-green value (a*) had no significant differences. These color values changed in response to convective drying conditions, with an immediate increase in paddy temperature caused by RF energy, which accelerated the Maillard reaction and the transfer of color substances from the rice husk and rice bran to the endosperm, causing discoloration [34,35,36,37]. However, considering HA as a conventional convective method in industrial drying of paddy, the WI value was used to compare the whiteness of white rice between treatments, and there was no significant difference at WI. On the other hand, ΔE* should be a single metric to compare the distance of color difference of HA /RF from HA. A lower ΔE* indicated greater accuracy, while a higher ΔE* indicated significant deviation. The ΔE* values in this study ranged from 1.25 ± 0.53 to 1.76 ± 0.26 and showed no significant difference. Therefore, it could be concluded that all RF heating temperatures in this study did not cause any color difference and were suitable for drying paddy on a large scale.

### 3.3. Effects on Cooking Qualities

#### 3.3.1. Elongation Ratio

Table 6 shows the elongation ratio (ER) of cooked rice after treatments. It was found that ER increased significantly with increasing RF heating temperature, as it was also observed in accelerated aged paddy in hot air oven [38], microwave [39], and RF [4,5]. This was due to the fact that RF energy could create fissures in the rice kernel, through which water could penetrate more easily into the rice kernel, and that after treatment, the rice had uniformly closed cell walls that resisted the high pressure inside the cell during cooking [39]. In addition, such heating also resulted in various changes in protein and starch granules that affected the swelling of the cooked rice kernel, as these two components were related to the water absorption of the kernel [40,41]. Although the length expansion without increasing the width of the rice kernel was considered a desirable property of rice, the result of the longest ER in HA/RF50 showed only 2.11% longer grain than the rice dried with HA, so it should not be important that the increase of ER was beneficial for the commercial aspect.

#### 3.3.2. Apparent Amylose Content

The apparent amylose content (AC) is shown in Table 6. It was found that there was no significant difference in AC of milled rice after treatment with RF compared to the control. This might be due to the fact that the temperatures of RF in this study were not too high to affect AC, as described by Wani et al. [42]; the H-bonds in amylose molecules can form inclusion complexes under high moisture and temperature conditions, and these H-bonds absorb more water and form clathrates with proteins and free fatty acids, which might be the reason for the decrease of AC in high moisture rice treated with RF. There were reports of AC decline in accelerated aged rice induced by RF that AC was negatively affected by moisture content, RF exposure time [3], and RF temperatures [9]. In addition, Jiao et al. [8] reported that exposure to RF of raw rice, brown rice, and milled rice at 50 °C for 5 min could cause a decrease in AC, while Hou et al. [7] argued that exposure to RF of raw rice, brown rice, and milled rice at 54 °C for 11 min showed no significant difference in AC. It could be considered that drying paddy with HA/RF at low temperature (38 °C–50 °C) could maintain the AC of rice as in HA.

#### 3.3.3. Texture Profiles Analysis

Table 7 shows the texture profiles of cooked rice after the treatments in terms of hardness, adhesiveness, springiness, and cohesiveness. Comparing the texture profiles of rice after RF heating and control, all the properties were not significantly different. This could mean that drying paddy with HA/RF at low temperature (38 °C–50 °C) could maintain the texture profiles of cooked rice as in HA.

#### 3.3.4. Pasting Properties

The pasting properties of cooked rice after the treatments in terms of peak viscosity, trough, breakdown, final viscosity, and setback are shown in Table 8. Comparing the pasting properties of rice after RF heating and control, all properties were not significantly different. It could be proposed that drying paddy with HA/RF at low temperature (38 °C–50 °C) could maintain the pasting properties of cooked rice as in HA.

### 3.4. Sensory Analysis

**Figure 5:**
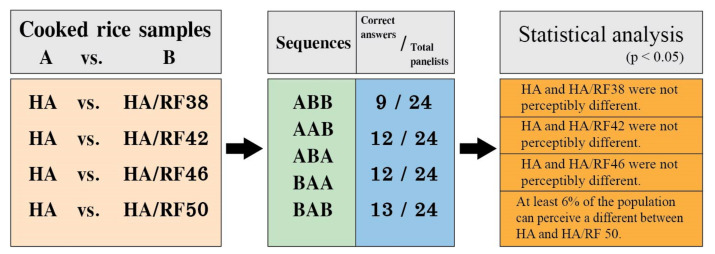
Triangle test to evaluate the ability of panelists to distinguish cooked rice dried with hot air (**A**) from cooked rice dried with hot air combined with the different RF heating temperatures (**B**). The statistical difference was determined using ISO 4120:2004 standardized tables.

A triangle test was conducted to determine whether panelists were able to distinguish cooked rice dried with hot air (control) from cooked rice dried with hot air combined with RF heating, as shown in Figure 5. It was found that cooked rice dried with HA/RF50 was significantly different from HA, and at least 6% of the population was able to perceive a difference. The proportion of the population able to perceive a difference between samples of cooked rice was calculated using a one-sided lower confidence interval, as shown in Equations (17)–(20):

(17) Proportion correct pc=xn

(18) $$\mathrm{Proportion}\;\mathrm{distinguished}\;\left(p_{d}\right)=1.5p_{c}-0.5$$

(19) $${\mathrm{Standard}\;\mathrm{deviation}\;\mathrm{of}\;p}_{d}\;\left(s_{d}\right)=1.5\sqrt{p_{c}\left(1-p_{c}\right)/n}$$

(20) $$\mathrm{Lower}\;\mathrm{confidence}\;\mathrm{limit}=p_{d}+z_{\alpha }s_{d}$$

where x is the number of correct responses, n is the total number of panelists, and z_α_ is the critical value of the standard normal distribution (z_α_ = 1.64 for a 95% confidence interval).

## 4. Conclusions

The effects on milling and cooking qualities of milled rice at four different RF heating temperatures (38 °C, 42 °C, 46 °C, and 50 °C) combined with hot air drying (HA/RF) were compared with hot air drying (HA). The drying characteristics and moisture diffusivity showed that the higher the RF heating temperature, the shorter the observed drying time. The amount of SEC and the specific energy cost decreased when the RF heating increased. In rice drying, milling qualities are the most important factors as they are the first stage of processing. A new rice drying technology must be proven to maintain the fissure percentage, the percentage of head rice yield, and the color of milled rice compared to conventional rice drying technology. Regarding milling qualities, the optimal condition for RF heating temperature was HA/RF42 in terms of fissure percentage, while there was no significant difference between all treatments in terms of head rice yield and white rice color determination. Regarding cooking qualities, drying paddy with HA/RF at the given temperature (38 °C–50 °C) showed no significant difference with HA in terms of amylose content, texture profiles, and pasting properties. Therefore, in this study, the cooking qualities were investigated to ensure that they were also maintained, while many previous studies stated that RF heating could change these qualities depending on the intensity of RF energy used, such as accelerated ageing of rice by RF heating. In the triangle test, it was found that at least 6% of the population could perceive a difference between HA and HA/RF50. In this study, it was proposed that HA/RF42 was the optimal condition for using a continuous flow hot air dryer in combination with radio frequency heating in a large-scale process. Compared to the previous study [12], there are three advantages of this significant improvement: (1) The fissure percentage on HA/RF42 was lower than on HA/RF45. The fissure percentage was the first parameter for a rice miller to determine the efficiency of the dryer. This result shows that lower RF heating temperature can better maintain the milled rice quality. (2) The power consumption of HA/RF42 could save up to 15.19% of the power of RF heating machine. The power consumption of the hot air dryer can be minimized by using various heat sources, such as liquefied petroleum gas (LPG), rice husk and biomass. (3) With a lower RF heating temperature, the RF heating machine can be redesigned to minimize the initial investment cost of combining with a small batch hot air dryer, or the RF heating machine can be combined with a batch dryer with larger drying capacity to generate more revenue.

## Figures and Tables

**Table 1:** Estimated values of the coefficients for selected thin-layer drying models and average effective moisture diffusivity (D_eff_) of combined RF heating with hot air drying.

Treatment	Lewis		Henderson–Pabis			Average (D_eff_ × 10^−9^ m^2^/s)
	k	R^2^	a	k	R^2^	
HA	1.3405 × 10^−^^3^	0.9702	8.6196	1.0373 × 10^−3^	0.9286	4.8156 ± 1.4550
HA/RF38	1.8522 × 10^−3^	0.9775	8.9515	1.5357 × 10^−3^	0.9391	6.2788 ± 2.3776
HA/RF42	1.9998 × 10^−3^	0.9733	8.7751	1.6038 × 10^−3^	0.9321	7.3994 ± 2.0088
HA/RF46	2.1854 × 10^−3^	0.9722	8.7203	1.7436 × 10^−3^	0.9297	9.0804 ± 1.6978
HA/RF50	2.6657 × 10^−3^	0.9776	8.9376	2.2164 × 10^−3^	0.9420	9.4426 ± 4.0670

Data of D_eff_ are expressed as mean ± SD in triplicate.

**Table 2:** Electricity consumption, specific energy consumption, and specific energy cost after being dried under various RF heating temperatures.

Treatment	Power Consumption (kWh)		Specific Energy Consumption	Energy Cost
	Hot Air Dryer	RF Heating Machine	(MJ/kg-H_2_O)	(USD/kg-H_2_O)
HA	59.33 ± 3.79 ^c^	0.00	4.0367 ± 0.6121	0.1098 ± 0.0167
HA/RF38	44.67 ± 4.04 ^b^	10.09 ± 1.15 ^a^	3.8700 ± 0.2265	0.1053 ± 0.0062
HA/RF42	40.67 ± 6.11 ^b^	15.81 ± 3.21 ^b^	3.9133 ± 0.4574	0.1065 ± 0.0124
HA/RF46	37.33 ± 7.51 ^b^	18.53 ± 3.05 ^b^	3.8600 ± 0.2261	0.1051 ± 0.0061
HA/RF50	28.67 ± 1.53 ^a^	24.36 ± 2.00 ^c^	3.7033 ± 0.4450	0.1009 ± 0.0121

Data are expressed as mean ± SD in triplicate. The same letter or no letter indicates no significant difference (*p* > 0.05) between the same values in a column.

**Table 3:** Effects of different RF heating temperatures on the fissure percentage of brown rice with different fissure degrees.

Treatment	None	Few	Moderate	Severe
	(0 Fissure)	(1–2 Fissures)	(3–4 Fissures)	(≥5 Fissures)
HA	99.78 ± 0.44 ^e^	0.33 ± 0.50 ^a^	0.00 ^a^	0.00 ^a^
HA/RF38	98.67 ± 0.50 ^d^	1.33 ± 0.50 ^b^	0.00 ^a^	0.00 ^a^
HA/RF42	96.33 ± 0.71 ^c^	3.89 ± 0.78 ^c^	0.00 ^a^	0.00 ^a^
HA/RF46	95.00 ± 0.87 ^b^	4.00 ± 0.87 ^c^	0.22 ± 0.44 ^ab^	0.78 ± 0.67 ^b^
HA/RF50	85.33 ± 1.87 ^a^	12.44 ± 1.81 ^d^	0.56 ± 0.73 ^b^	1.78 ± 0.83 ^c^

Data are expressed as mean ± SD in triplicate. The same letter or no letter indicates no significant difference (*p* > 0.05) between the same values in a column.

**Table 4:** Effects of different RF heating temperatures on the milling yield percentage.

Treatment	Husk	Brown Rice	White Rice	Head Rice	Broken Rice	Bran
HA	25.33 ± 0.40 ^b^	74.67 ± 0.40 ^a^	65.07 ± 0.80 ^b^	38.40 ± 2.65	26.67 ± 2.00 ^a^	9.60 ± 0.89 ^a^
HA/RF38	25.60 ± 0.57 ^b^	74.40 ± 0.57 ^a^	63.82 ± 0.96 ^a^	37.51 ± 2.73	26.31 ± 1.94 ^a^	10.58 ± 1.12 ^ab^
HA/RF42	25.42 ± 0.78 ^b^	74.58 ± 0.78 ^a^	64.44 ± 1.21 ^ab^	38.22 ± 1.69	26.22 ± 1.37 ^a^	10.13 ± 0.69 ^ab^
HA/RF46	25.78 ± 0.67 ^b^	74.22 ± 0.67 ^a^	63.64 ± 1.50 ^a^	37.33 ± 2.62	26.31 ± 1.47 ^a^	10.58 ± 1.59 ^ab^
HA/RF50	24.62 ± 0.78 ^a^	75.38 ± 0.78 ^b^	64.62 ± 1.25 ^ab^	36.62 ± 1.87	28.00 ± 1.79 ^b^	10.76 ± 0.81 ^b^

Data are expressed as mean ± SD in triplicate. The same letter or no letter indicates no significant difference (*p* > 0.05) between the same values in a column.

**Table 5:** Effects of different RF heating temperatures on the white rice color.

Treatment	L*	a*	b*	Whiteness Index	ΔE*
HA	64.51 ± 0.69	−0.54 ± 0.14 ^a^	9.02 ± 0.51 ^a^	63.37 ± 0.65	0.00
HA/RF38	64.15 ± 1.02	−0.41 ± 0.27 ^ab^	9.95 ± 1.13 ^b^	62.78 ± 1.24	1.28 ± 1.08
HA/RF42	64.07 ± 1.89	−0.41 ± 0.27 ^ab^	9.75 ± 1.14 ^ab^	62.76 ± 2.06	1.76 ± 1.26
HA/RF46	64.01 ± 0.81	−0.36 ± 0.32 ^b^	10.09 ± 0.41 ^b^	62.61 ± 0.81	1.41 ± 0.51
HA/RF50	64.61 ± 1.02	−0.38 ± 0.29 ^b^	9.43 ± 0.74 ^ab^	63.37 ± 0.94	1.25 ± 0.53

Data are expressed as mean ± SD in triplicate. The same letter or no letter indicates no significant difference (*p* > 0.05) between the same values in a column.

**Table 6:** Effects of different RF heating temperatures on the elongation ratio and apparent amylose content.

Treatment	Elongation Ratio	Apparent Amylose Content (%)
HA	1.4257 ± 0.0193 ^a^	26.74 ± 0.24
HA/RF38	1.4264 ± 0.0228 ^a^	27.76 ± 0.94
HA/RF42	1.4377 ± 0.0169 ^b^	27.07 ± 0.86
HA/RF46	1.4533 ± 0.0291 ^c^	26.75 ± 0.87
HA/RF50	1.4530 ± 0.0196 ^c^	27.68 ± 0.37

Data are expressed as mean ± SD in triplicate. The same letter or no letter indicates no significant difference (*p* > 0.05) between the same values in a column.

**Table 7:** Effects of different RF heating temperatures on the textural profiles of cooked rice.

Treatment	Hardness (g)	Adhesiveness (g.sec)	Springiness (sec/sec)	Cohesiveness (g.sec/g.sec)
HA	10,400.28 ± 253.68	−145.89 ± 77.63	0.6973 ± 0.0151	0.4599 ± 0.0029
HA/RF38	10,160.72 ± 610.38	−108.92 ± 24.25	0.6560 ± 0.0354	0.4548 ± 0.0069
HA/RF42	9828.77 ± 109.48	−123.28 ± 68.41	0.6868 ± 0.0470	0.4536 ± 0.0064
HA/RF46	10,045.50 ± 337.98	−99.91 ± 69.41	0.6736 ± 0.0374	0.4572 ± 0.0223
HA/RF50	10,340.02 ± 168.02	−122.87 ± 56.59	0.6500 ± 0.0361	0.4560 ± 0.0042

Data are expressed as mean ± SD in triplicate. The same letter or no letter indicates no significant difference (*p* > 0.05) between the same values in a column.

**Table 8:** Effects of different RF heating temperatures on the pasting properties of cooked rice.

Treatment	Peak Viscosity (cP)	Trough (cP)	Breakdown (cP)	Final Viscosity (cP)	Setback (cP)
HA	1474.78 ± 124.60	982.33 ± 108.89	492.44 ± 24.39	2195.45 ± 162.07	720.67 ± 38.11
HA/RF38	1591.33 ± 141.81	1028.89 ± 121.74	562.44 ± 24.93	2306.78 ± 200.30	715.44 ± 58.51
HA/RF42	1503.78 ± 48.10	970.56 ± 32.08	533.22 ± 76.42	2208.45 ± 92.69	704.67 ± 138.85
HA/RF46	1595.89 ± 258.83	1024.78 ± 137.36	571.11 ± 121.47	2266.33 ± 231.49	670.45 ± 32.70
HA/RF50	1768.22 ± 220.94	1068.00 ± 93.02	700.22 ± 127.95	2372.00 ± 88.76	603.78 ± 133.52

Data are expressed as mean ± SD in triplicate. The same letter or no letter indicates no significant difference (*p* > 0.05) between the same values in a column.

## Data Availability

Not applicable.
